# Maladaptive personality traits as predictors of prosocial and trusting behavior in two economic games

**DOI:** 10.1186/s40479-022-00201-0

**Published:** 2022-11-10

**Authors:** Johanna Hepp, Melissa R. M. Mohr, Inga Niedtfeld

**Affiliations:** 1 Department of Psychosomatic Medicine and Psychotherapy, Central Institute of Mental Health, Medical Faculty Mannheim/Heidelberg University, J5, 68159 Mannheim, Germany; 2 University of Mannheim, Mannheim, Germany

**Keywords:** Maladaptive personality traits, Personality disorder, Antagonism, Suspiciousness, Prosocial behavior, Trust, Economic games, Dictator game, Faith game

## Abstract

**Background:**

Dimensional models of personality disorders postulate interpersonal dysfunction as the core feature of personality pathology, and describe maladaptive personality traits that characterize the specific pattern of dysfunction that is experienced. Herein, we examined whether maladaptive traits predict prosocial and trusting behavior, both of which are highly relevant behaviors for interpersonal functioning. Specifically, we examined antagonism as a predictor of prosocial behavior in a dictator game, and suspiciousness as a predictor of trust in the faith game.

**Materials and methods:**

The study was preregistered and conducted online. The preregistration protocol is available at https://osf.io/er43j. Data and code are available at https://osf.io/2rvbg/. Participants (*N* = 445) completed the German version of the Personality Inventory for DSM-5 to measure antagonism and suspiciousness. Additionally, they played the dictator game (more money taken away from another person indicates less prosocial behavior) and the faith game (choosing the sure choice instead of the faith choice indicates less trust). We conducted a linear regression model to test whether antagonism is associated with prosocial behavior in the dictator game and a logistic regression model to test whether suspiciousness predicts selection of the sure choice in the faith game.

**Results:**

As hypothesized, higher levels of antagonism were associated with less prosocial behavior in the dictator game. The remaining hypotheses were not supported, as suspiciousness was not significantly associated with the likelihood of choosing the sure choice in the faith game. Exploratory analyses on participants’ estimates of the sure choice amount suggest successful experimental manipulation in the faith game.

**Conclusions:**

The results on antagonism and prosocial behavior are consistent with those of previous studies that used categorial classification systems of personality disorders or examined non-pathological personality traits. Potential explanations for the non-significant effects of suspiciousness are discussed, including the small size and range of the sure choice payoff and that the anonymity of the game may have precluded suspicious traits from expressing. Future research with higher stakes and known interaction partners is needed to further probe the effects of suspiciousness.

**Supplementary Information:**

The online version contains supplementary material available at 10.1186/s40479-022-00201-0.

## Introduction

With the inclusion of the alternative model for personality disorders (AMPD) in the fifth edition of the diagnostic and statistical manual of mental disorders (DSM-5; [1]) and the fully dimensional personality disorder (PD) diagnosis in ICD-11 [2], the diagnosis of PD has undergone a paradigm shift from categorical to dimensional systems. While the AMPD still comprises a hybrid model that combines categorical and dimensional aspects, the ICD-11 model is fully dimensional, allowing for the specification of different levels of PD severity. Both approaches postulate *interpersonal dysfunction* and *self-related dysfunction* as the core criteria for PD and detail maladaptive personality traits for further specification of the diagnosis. ICD-11 allows for the optional specification of five maladaptive trait qualifiers, whereas the AMPD requires that individuals must meet for one or more maladaptive traits. Both systems describe largely overlapping traits of *negative affectivity* (the tendency to experience a broad range of negative emotions), *detachment* (the tendency to maintain interpersonal and emotional distance), *antagonism* (DSM-5)/ *dissociality* (ICD-11) (the tendency to disregard the rights and feelings of others), and *disinhibition* (the tendency to act rashly and without consideration of potential negative consequences). DSM-5 further specifies a *psychoticism* trait (the tendency for unusual beliefs and experiences, eccentricity, and cognitive and perceptual dysregulation), whereas ICD-11 includes *anankastia* as a fifth trait (the tendency to control one’s own behavior, the behavior of others, and situations to ensure conformity to rigid standards).

Multiple personality theories postulate that personality traits express themselves more or less strongly depending on the situational affordance that is given, and thus conceptualize behavior as the outcome of a person×situation interaction (e.g., see [3] for an overview). Based on a previous literature review [4], we identified prosocial and trusting behavior as two central components of interpersonal functioning that are impaired in PDs and that likely markedly contribute to interpersonal dysfunction. When individuals do not show prosocial behavior towards others (i.e. “voluntary, intentional behavior that results in benefits for another”, ([5], p. 92)), this may impede both the formation and maintenance of relationships. Interaction partners will be unlikely to engage with someone who does not behave pro-socially toward them (e.g. by doing them a favor, showing acts of kindness) and may eventually turn away from the person and end the relationship. In turn, individuals who show levels of trusting behavior may themselves be unlikely to form and maintain relationships, as they do not expect others to behave benevolently toward them.

In the present study, we investigate whether maladaptive traits are predictive of interpersonal dysfunction (a core criterion for PD in both systems) in the presence of specific situational affordances. In their *affordance framework of prosocial behavior,* Thielmann et al. [6] detail the situational characteristics that are required for personality traits to manifest in prosocial and trusting behavior. Overall, their framework specifies four situational affordances: the possibility for exploitation (i.e., the power to enrich oneself at the expense of others without having to fear negative consequences), the possibility of reciprocity (i.e., the ability to respond to or reciprocate a person’s previous behavior), a temporal conflict between short- and long-term interests (i.e., the need to consider how one’s actions in the present will affect future outcomes), and dependence under uncertainty (i.e., making a decision without knowing how the interaction partners will act or react). The two situational affordances we focus on herein are the possibility for exploitation, under which prosocial behavior can be shown or not shown, and dependence under uncertainty, under which trusting behavior can be shown or not shown.

Based on the trait and facet-level content of the maladaptive personality traits postulated for DSM-5 AMPD, we identified the antagonism trait (dissociality in ICD-11) as a likely predictor for low prosocial behavior when given the possibility for exploitation, and the detachment facet suspiciousness for low trusting behavior in situations with dependence under uncertainty [4]. This selection was based on both considerations of the trait/facet level content, which theoretically implies predictiveness for prosocial or trusting behavior and on previous evidence linking related constructs to these behaviors. *Antagonism* encompasses callous antipathy toward others and unawareness of others’ needs and feelings, as well as a willingness to use others in the service of self-enhancement [1]. This suggests that individuals high on this trait may show a tendency to use others to their own advantage, when given the opportunity. Previous work supports this, as individuals with a categorical diagnosis of antisocial personality disorder, who score high on antagonism [7], showed lower prosocial behavior in the *dictator game* [8, 9]. The dictator game [10, 11] is an economic game that provides an opportunity for exploitation. Participants playing the role of the ‘dictator’ can subtract money from an interaction partner without negative consequences for themselves (i.e. exploit the other person or behave prosocially toward them). In previous studies, participants with antisocial personality disorder subtracted higher amounts of money from interaction partners than control participants did. Likewise, individuals who score high on five-factor model [12] agreeableness (the lower end of which reflects antagonism) showed greater prosocial behavior (sharing more money) in the dictator game [13, 14], as did individuals high on HEXACO [15] honesty-humility [16, 17]. Taken together, this suggested that translated - to a maladaptive trait framework - we antagonism should be associated with lower levels of prosocial behavior in the dictator game. Consequently, we hypothesized: The level of antagonism is positively associated with the amount of money participants subtract from recipients in the dictator game (hypothesis 1).

*Suspiciousness* is a facet of the maladaptive trait detachment and encompasses mistrust and doubt with respect to the loyalty and fidelity of others [1]. Individuals loading high on this trait believe that other people will exploit or harm them if given the opportunity. This suggests that individuals with high levels of suspiciousness may avoid situations in which they would have to trust the fairness of others, if they can choose a safer alternative. Previous work supports this, as individuals with a categorical diagnosis of borderline personality disorder, who score high on suspiciousness [18, 19], showed low levels of trust in general [20, 21], and low levels of trust in situations characterized by dependence under uncertainty [22]. To test the association between suspiciousness and trust under uncertainty, one can employ the *faith game* [23]. In this game, participants can choose between a fixed amount of money given to them by the experimenter (sure choice) and an unknown amount of money (faith choice) left for them by an unknown person. The amount of money presented as the sure choice can make it more or less attractive to participants.

Based on the definition of the suspiciousness facet and previous findings on borderline personality disorder, we hypothesized that the level of suspiciousness is positively associated with the likelihood of selecting the sure choice over the faith choice in the faith game (Hypothesis 2). Additionally, we expected that the amount offered as the sure choice is positively associated with the likelihood of selecting the sure choice over the faith choice (Hypothesis 3). Lastly, we hypothesized that the association between the amount of money offered as the sure choice and the likelihood of selecting the sure choice is moderated by the level of suspiciousness. More specifically, we expected individuals high in suspiciousness to have a strong overall preference for the sure choice, thus their decision should be influenced less strongly by the exact amount offered as the sure choice. They should be more likely to select the sure choice even when the sure choice amount is small. In other words, individuals high in suspiciousness would rather accept a small amount of money that they will definitely receive (sure choice) than rely on someone else to share with them (faith choice). Accordingly, we hypothesized that the level of suspiciousness weakens the association between the amount of money offered as the sure choice and the likelihood of selecting the sure choice over the faith choice in the faith game (Hypothesis 4).

## Method

We conducted the study in German language and online, hosted on the platform SoScisurvey (www.soscisurvey.de; [24]) from February 2 to March 3, 2022. The study design and methods were preregistered, and the protocol is available at https://osf.io/er43j. Study procedures were approved by the ethics committee of the medical faculty Mannheim at Heidelberg University (protocol no. 2021–524). All participants provided informed consent prior to their participation.

### Participants

We advertised the study as investigating the association between personality traits and decision-making. As we wanted to assess a wide range of maladaptive trait levels, including participants with high maladaptive trait levels, we recruited through Facebook groups, Instagram pages, and online forums dedicated to personality disorders and mental health topics in general. Additionally, we sent invitation emails to individuals that participated in previous studies on borderline personality disorder and childhood maltreatment within our clinic. Additionally, we recruited participants via student mailing lists at the University of Mannheim. Based on a-priori power-analyses, we pre-registered a requited sample size of *N* = 390 (for details, see pre-registration protocol at https://osf.io/er43j). Since we pre-registered a number of data exclusion criteria (e.g., repeated participation, careless participation) to ensure data quality, we planned to oversample by approximately 15%. Eventually, a total of 460 individuals participated in the online study. Based on pre-registered exclusion criteria, we excluded nine participants (1.96% of the sample) because they spent less than 2 seconds on answering each item of the PID-5 questionnaire, four participants (0.87%) because they indicated that we should not use their data due to bad quality, and two participants (0.43%) because of repeated participation.

After applying the exclusion criteria, the final sample comprised 445 participants. Of these, 253 participants (56.85%) were recruited via advertisements in Facebook groups, online forums, Instagram channels, and websites dedicated to mental health topics. In addition, 81 participants (18.20%) who had participated in previous studies of borderline personality disorder and childhood maltreatment in our clinic were recruited via mailing lists. The remaining 111 participants (24.94%) were recruited via advertising in non-clinical settings (i.e., University of Mannheim mailing lists and snowball method on social media). The age of participants in the final sample ranged from 18 to 73 years (*M* = 33.79, *SD* = 13.17). The diversity of the sample was limited in terms of gender, nationality, and migration background, as most participants were women (*n* = 335, 75.28%), had a German nationality (*n* = 427, 95.96%), and had no migration background (*n* = 374, 84.04%). Furthermore, most participants had a university entrance qualification or university degree (*n* = 289, 64.99%) and were currently employed (*n* = 180, 40.45%) or studying (*n* = 112, 25.27%). Almost all participants were native German speakers (*n* = 431, 96.85%). Participants indicated an average monthly net income of 1509.00€ (*SD* = 1344.94), ranging from 0€ to 9500€. A total of 54 participants (12.13%) identified as belonging to a minority group (in detail, 12 participants (2.70%) due to belonging to the queer community, 9 participants (2.02%) due to mental disorders, 5 participants (1.12%) due to religion and 28 participants (6.29%) due to other reasons). Detailed demographic data are shown in Table 1.

**Table 1:** Sociodemographic characteristics of the sample

	*N* = 445	
	*n*	%
Gender identity		
Female (cisgender)	334	75.06
Male (cisgender)	88	19.78
Female (transgender)	1	0.22
Male (transgender)	5	1.12
Non-binary	7	1.57
Agender	5	1.12
Bigender	1	0.22
Did not want to indicate their gender	4	0.90
Highest education level		
No degree	3	0.67
Still in school	8	1.80
Graduated after 9 years of education	17	3.82
Graduated after 10 years of education	42	9.44
University entrance level degree	169	37.98
Completed apprenticeship	50	11.24
Bachelor’s degree	50	11.24
Master’s degree/diploma	78	17.53
Master craftsman	6	1.35
PhD	17	3.82
Did not want to indicate their educational level	5	1.12
Nationality		
German	427	95.96
Other	18	4.04
Employment		
Unemployed	10	2.25
Pupil	10	2.25
Apprenticeship	16	3.60
Student	112	25.17
Employee	180	40.45
Self-employed	21	4.72
Civil servant	10	2.25
On long-term sick leave	33	7.42
Retired	30	6.74
Other	15	3.37
Did not want to indicate their employment	8	1.80
Migration background		
No migration background	374	84.04
First-generation migrant	9	2.02
Second-generation migrant	42	9.44
Third-generation migrant	15	3.37
Did not want to indicate	5	1.12
Language level (German)		
Native	431	96.85
Fluent	7	1.57
Very good	4	0.90
Good	3	0.67

Regarding clinical characteristics, 202 participants (45.39%) indicated that they had been diagnosed with at least one mental disorder in the past. The most frequently self-reported diagnoses were mood disorders (*n* = 120, 26.97%) and personality disorders (*n* = 91, 20.45%). Details of these self-reported clinical characteristics are shown in Table 2.

**Table 2:** Self-reported Diagnoses of Participants (Mental Disorders)

	*N* = 445	
	*n*	%
Personality disorder	91	20.45
Borderline personality disorder	77	17.30
Post-traumatic stress disorder	64	14.38
Anxiety disorder	42	9.44
Mood disorder	120	26.97
Eating disorder	24	5.39
Other	67	15.06

Of all participants, 175 indicated their specific diagnoses. Of these 175 participants, 135 participants (77.14%) indicated more than one diagnosis

### Procedure

After providing demographic information, participants were asked to complete the German version of the PID-5 [25] and the Level of Personality Functioning Scale-Brief Form (LPFS-BF, [26]; the LPFS-BF data will be used as pilot data for another project. Next, they participated in two economic games – either the *dictator game* or the *faith game*, in randomized order. We implemented five versions of the faith game with varying sure choice pay-offs, and each participant played one version that was randomly selected. After completing the first game, participants rated a series of positive and negative interpersonal statements that served as a filler for the gap between the two games and that will be used in a different project. Following this, participants completed the second game. If participants played the faith game first, they were not informed about the payoff of the faith choice (if they chose it) until after they had completed the dictator game, to avoid spillover effects.

Both games were partially incentivized. This means that, for some participants, we actually paid out the money they earned in the dictator game and the faith game at the end of the study via an amazon voucher. The voucher was automatically displayed on the last study page, so complete anonymity was guaranteed to participants (i.e., they did not have to contact the experimenter or leave any identifying information to receive the voucher). Participants were informed that “every 10th player” would win their actual outcome. Randomly, 47 participants (10.22%) were selected to receive their actual payoff. This payoff could vary between 2€ (if participants only kept their initial endowment in the dictator game and received 0€ in the faith game, when choosing the faith choice) and 18€ (if they took all the money in the dictator game, thus earning 10€ there, and if they received 8€ via the faith choice in the faith game).

### Materials

#### Personality inventory for DSM-5

We assessed maladaptive personality traits using the German version of the PID-5 [25]. In detail, we used the translation of the 100-item short form (PID-5-SF, [27]), which covers 25 maladaptive personality trait facets nested within the five DSM-5 maladaptive trait domains negative affectivity, detachment, antagonism, disinhibition, and psychoticism. To test the hypotheses described above, we used the domain score for antagonism, which is a mean score calculated for all items within this dimension (based on 24 items). As the 100-item short form includes only four items for the PID-5 facet suspiciousness, we added three additional items for suspiciousness taken from the 220-item long form of the PID-5 and calculated the mean score for suspiciousness including all seven items. Internal consistencies for the PID-5-SF dimension antagonism (α = 0.90) and PID-5 facet suspiciousness (α = 0.89) were high in this sample.

#### Dictator game

In the dictator game [10], participants (in the role of the allocator, player A) played a hypothetical game for money with an unknown interaction partner (player B). Each participant was informed that a subsequent participant in the same study would assume the role of player B, and that the exchange would be completely anonymous. In the present study, we used a modified dictator game (i.e., the instructions differed somewhat from the classic implementation of the game as described by Forsythe et al., [10]), previously used by List [11]. Participants were informed that they would play the role of the allocator (player A). They received 2€ by the experimenter as an initial budget, and we informed them that the other person (player B) received 8€. Participants were then told that they could take as much as they wanted from player B’s money stash and keep it for themselves (in addition to their own 2€). They were also informed that player B would have no way to react to their decision and that both player A and B would remain completely anonymous. In the role of player A, they then decided which amount of money they wanted to subtract from player B’s money stash (0€-8€). The amount of money participants deducted from player B’s money stash was used as an indicator of prosocial behavior (inverted, i.e., higher amounts taken away indicated higher levels of selfish behavior / lower prosociality). The maximum amount of money participants could earn in this game was 10€ (if they subtracted the entire 8€ from the recipient), and the minimum amount was 2€ (if they took nothing from the recipient and kept only their initial endowment). The dictator game did not include any within- or between-person conditions.

#### Faith game

In the faith game [23], participants were assigned the role of player B. They were informed that a previous participant had been in the role of player A and left money in an envelope for player B. Participants (in the role of player B) did not know the amount of money that player A had left for them (the amount could be between 0€ and 8€). Participants were asked to decide whether they wanted to receive the amount that player A had left for them (faith choice) or whether they wanted to receive a fixed amount of money from the experimenter (sure choice). These fixed amounts were randomly varied at five levels (2€, 2.5€, 3€, 3.5€, 4€) as a between-participants factor and participants were shown the amount they would receive from the experimenter if they chose this option (i.e. they were aware of the amount of the sure choice). The resulting outcome variable is dichotomous and indicates whether the participant chose the sure choice or the faith choice. Choosing the sure choice indicates less trust because of lower trust in the prosociality of others. The maximum amount of money participants could receive was 8€ (if they chose the faith choice and player A had left the entire 8€ to them), the minimum amount of money they could receive was 0€ (if they chose the faith choice and player A had left them 0€). For exploratory analyses, participants were asked to guess the amount of money in the envelope of the faith choice (amount of money that a previous participant left for them).

### Data analysis

All data and analysis code is available at https://osf.io/2rvbg/. Data were analyzed using R [28] and all analyses were pre-registered (https://osf.io/er43j). To test our hypotheses, we conducted a linear multiple regression and a logistic regression model. In all models, we adjusted for the pre-registered covariate gender (dummy-coded, 0 for women [trans and cis] and 1 for men [trans and cis]), because meta-analytic results suggest cooperative behavior may vary as a function of gender [29]. All analyses were calculated on a sample of *n* = 428 participants, because the remaining 17 participants did not indicate their gender identity or reported a gender identity other than cis/trans male/female.^1^

To test H1, we entered the PID-5-SF dimension antagonism (mean score) and the covariate gender into a linear regression model to predict prosocial behavior in the dictator game (i.e., the amount of money taken). To test H2, H3, and H4, we conducted a logistic regression model with the likelihood of choosing the sure choice (versus the faith choice) as outcome. Choice was coded as 1 for the sure choice and 0 for the faith choice. As predictors, the model included the PID-5 facet suspiciousness (centered on the grand mean) and the amount of money offered as the sure choice (varying between-participants: 2€, 2.50€, 3€, 3.50€, 4€, centered on the grand mean), as well as their interaction, and the covariate gender. The main effect of suspiciousness constitutes the test of H2. The main effect of the amount of money offered as the sure choice constitutes the test of H3. The interaction term constitutes the test of H4.

## Results

### Descriptive results

**Fig. 1:**
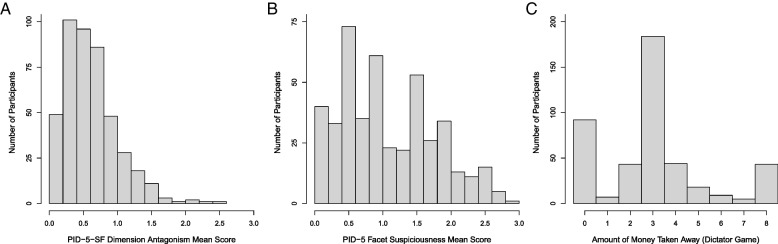
Histograms of the Mean Scores of the PID-5-SF Dimension Antagonism (**A**), Mean Scores of the PID-5 Facet Suspiciousness (**B**), and the Amounts of Money Taken Away in the Dictator Game (**C**)

The mean scores of the PID-5 dimension antagonism ranged from 0 to 2.54 (*M* = 0.61, *SD* = 0.40), with 0 indicating a low level and 3 indicating a high level of antagonism. Compared with the normal distribution, the distribution of antagonism mean scores was highly right-skewed (*skewness* = 1.19) and leptokurtic (*kurtosis* = 2.09). Figure 1A illustrates the distribution of the antagonism mean scores. The mean scores of the PID-5 facet suspiciousness ranged from 0 to 3 (*M* = 1.08, *SD* = 0.70), with 0 indicating a low level and 3 indicating a high level of suspiciousness. Compared with the normal distribution, the distribution of suspiciousness mean scores was right-skewed (*skewness* = 0.43) and platykurtic (*kurtosis* = − 0.72). Figure 1B illustrates the distribution of the suspiciousness mean scores.

The amount of money that participants took from player B in the dictator game ranged between 0€ an 8€ (*M* = 3.02€, *SD* = 2.25€). Compared to the normal distribution, the distribution of money taken away in the dictator game was right-skewed (*skewness* = 0.67) and leptokurtic (*kurtosis* = 0.20). A total of 184 participants (41.35%) divided the amount of money equally between themselves and player B (5€ each). In contrast, 92 participants (20.67%) took nothing from player B, whereas 43 participants (9.66%) took everything from player B. Figure 1C illustrates the distribution of the amount of money taken in the dictator game. In the faith game, 219 participants (49.21%) chose the sure choice, whereas 226 participants (50.79%) chose the faith choice. Detailed information on the number of participants per group and percentage of decisions per group can be found in Table 3.

**Table 3:** Descriptive statistics of the faith game

Sure choice amount	2.00€	2.50€	3.00€	3.50€	4.00€
Sure choice (%)	29.70	36.78	43.84	59.09	76.04
Faith choice (%)	70.30	63.22	56.16	40.91	23.96
Total number of participants	101	87	73	88	96

*Sure choice amount* Amount of money offered as sure choice in the faith game

### Confirmatory analyses

**Fig. 2:**
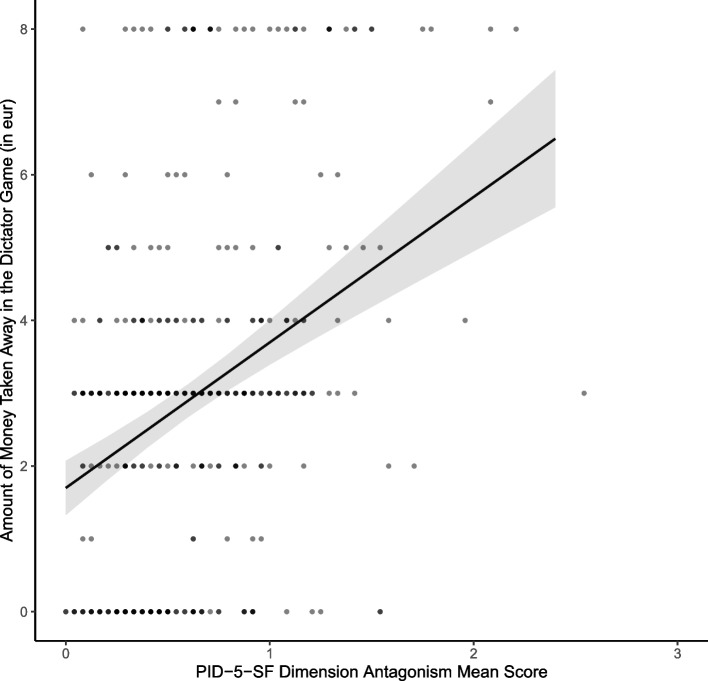
Regression Plot of the Predicted Main Effect of the Mean Score of the PID-5-SF Dimension Antagonism on the Amount of Money Taken Away in the Dictator Game (H1), Controlling for Gender. *Note.* The error band displays the 95% confidence interval. Additionally, raw data points are plotted

We conducted all confirmatory analyses as pre-registered. Supporting H1, the main effect of antagonism on dictator game contributions, adjusted for gender, was significant and positive, *b* = 2.00, β = 0.35, *p* < .001. This indicated that higher levels of antagonism were associated with less prosocial behavior in the dictator game (more money taken away from player B). Cohen’s *f*^*2*^ for the main effect of antagonism was 0.15, indicating a medium effect size [30]. The covariate gender was not significant, *b* = 0.37, β = 0.07, *p* = .138. A model plot for the multiple regression analysis is shown in Fig. 2.

The results for the test of H2, H3 and H4 are shown in Table 4. Contrary to our hypotheses, neither suspiciousness nor the amount of money offered as the sure choice showed a significant association with the likelihood of choosing the sure choice in the faith game. The interaction term of suspiciousness and the sure choice amount was also non-significant. Thus, hypotheses 2 to 4 were not supported.

**Table 4:** Logistic Regression Analysis for H2-H4: Effects of the Mean Score of the PID-5 Facet Suspiciousness and the Sure Choice Amount in the Faith Game on the Likelihood of Choosing the Sure Choice, adjusted for Gender

Effect	Estimate	*OR*	95% CI		*p*
			*LL*	*UL*	
Intercept	0.03	1.03	0.83	1.28	.759
Suspiciousness	0.06	1.07	0.81	1.41	.647
Sure choice amount	0.10	1.10	0.96	1.26	.166
Suspiciousness×sure choice amount	0.08	1.08	0.90	1.30	.409
Gender ^a^	−0.25	0.78	0.49	1.24	.296

*n* = 428

*CI* Confidence interval, *LL* Lower limit, *UL* Upper limit, *OR* Odds ratio, *Sure choice amount* Amount of money offered as sure choice in the faith game

^a^This variable is dummy-coded (0 for female, 1 for male)

### Exploratory analyses for the faith game

As described above, the pre-registered analyses for the faith game revealed that suspiciousness did not show a significant association with selecting the sure choice in the faith game. To further probe possible effects of suspiciousness, we conducted an exploratory analysis with participants’ estimates of the amount of money the other player left for them as the faith choice. In detail, we used a linear regression model with suspiciousness as a predictor of participants’ estimates of the faith choice amount, adjusted for gender. Results showed a significant and negative main effect of suspiciousness (*b* = − 0.27, β = −.10, *p* = .042), indicating that higher levels of suspiciousness were associated with lower estimates of the faith choice amount, though this effect was small *f*^*2*^ = 0.02.

Additionally, we conducted an exploratory analysis to further probe whether the faith game manipulation had the desired effect. For this purpose, we conducted a logistic regression model with the likelihood of choosing the sure choice, predicted by the faith choice estimate, the sure choice amount, and their interaction (adjusted for the covariate gender). Results showed a significant and negative main effect of the faith choice estimate (Est. = − 1.05, OR = 0.35, *p* < .001). That is, the higher the payoff participants expected as the faith choice, the more likely they were to select the faith choice. In addition, the interaction between the faith choice estimate and the sure choice amount was significant (Est. = 0.19, OR = 1.21, *p* = .041), indicating that participants were particularly prone to select the sure choice when the sure choice amount was high and their expectation of the faith choice amount was low. A regression plot illustrating the interaction term of the logistic regression analysis is shown in the online supplement, Fig. S1.

### Exploratory analyses for other PID-5 dimensions and facets

Based on the helpful suggestion of an anonymous reviewer, we conducted additional exploratory analyses in order to probe the specificity of the antagonism-dictator-game association and to investigate potential effects of the other PID-5 dimensions. Table 5 shows the results from a linear regression model using all PID-5 dimension scores simultaneously to predict dictator game extractions. Antagonism was the only significant, positive predictor of dictator game extractions, whereas detachment, negative affectivity, psychoticism, and disinhibition were not significantly associated with prosocial behavior in the game. Thus, the positive association between antagonism and dictator game contributions that we observed in the confirmatory analysis reported above replicated when testing whether antagonism shows incremental effects beyond the other PID-5 dimensions.

**Table 5:** Multiple Linear Regression Analysis for Replication of H1: Effects of the Mean Score of the PID-5-SF Dimensions on Prosocial Behavior in the Dictator Game (Amount of Money Taken Away), Adjusted for Gender

Effect	*b*	*SE*	*t*	β	95% CI		*p*
					*LL*	*UL*	
Intercept	1.63	0.33	1.98				
Antagonism	1.90	0.30	6.31	.34	0.23	0.44	< .001
Detachment	0.23	0.24	0.95	.06	−0.07	0.20	.345
Negative Affectivity	−0.30	0.24	−1.25	−.08	−0.21	0.05	.212
Psychoticism	−0.15	0.25	−0.58	−.04	−0.17	0.09	.560
Disinhibition	0.40	0.31	1.30	.07	−0.04	0.18	.193
Gender ^a^	0.26	0.26	0.99	.05	−0.05	0.14	.325

*n* = 428

*SE* Standard error, *CI* Confidence interval, *LL* Lower limit, *UL* Upper limit

*R*^*2*^ = 0.14. *F*(6, 421) = 11.64, *p* < .001

^a^This variable is dummy-coded (0 for female, 1 for male)

In a further exploratory analysis, we investigated whether any of the PID-5 dimensions show significant associations with the faith game performance (where the suspiciousness facet did not). Yet, as shown in Table 6, none of the PID-5 dimensions were significantly associated with the likelihood of choosing the sure choice in the faith game. Additionally, we performed a model in which we included suspiciousness and the other facets of the detachment dimension (anhedonia, depressivity, intimacy avoidance, restricted affectivity, and withdrawal) to predict faith game performance simultaneously. Again, suspiciousness did not show the hypothesized positive effects on the likelihood of selecting the sure choice. Only withdrawal showed a significant, albeit very small positive association with the behavior. However, this effect did not remain significant when adjusting the α-level for multiple testing of the eight predictors in the model, with the adjusted α for the withdrawal effect = .00625 (following [31]) (Table 7).

**Table 6:** Logistic Regression Analysis for Replication of H2-H4: Effects of the Mean Score of the PID-5 Dimensions and the Sure Choice Amount in the Faith Game on the Likelihood of Choosing the Sure Choice, Adjusted for Gender

Effect	Estimate	*OR*	95% CI		*p*
			*LL*	*UL*	
Intercept	0.54	1.71	0.92	3.23	.092
Sure choice amount	0.18	1.20	0.92	1.58	.188
Antagonism	−0.49	0.62	0.34	1.09	.100
Detachment	0.32	1.38	0.87	2.19	.167
Negative Affectivity	−0.41	0.66	0.41	1.05	.084
Psychoticism	0.45	1.56	0.97	2.55	.069
Disinhibition	−0.29	0.75	0.41	1.34	.329
Gender ^a^	−0.24	0.79	0.48	1.29	.346

*n* = 428

*CI* Confidence interval, *LL* Lower limit, *UL* Upper limit, *OR* Odds ratio; Sure choice amount = amount of money offered as sure choice in the faith game

^a^ This variable is dummy-coded (0 for female, 1 for male)

**Table 7:** Logistic Regression Analysis for Replication of H2-H4: Effects of the Mean Score of the Facets of the PID-5 Dimension Detachment and the Sure Choice Amount in the Faith Game on the Likelihood of Choosing the Sure Choice, adjusted for Gender

Effect	Estimate	*OR*	95% CI		*p*
			*LL*	*UL*	
Intercept	−0.18	0.84	0.56	1.26	.394
Sure choice amount	0.16	1.18	0.77	1.80	.452
Suspiciousness	−0.16	0.86	0.57	1.28	.449
Anhedonia	−0.19	0.83	0.52	1.31	.413
Depressivity	0.06	1.06	0.69	1.63	.778
Intimacy Avoidance	−0.08	0.92	0.69	1.24	.600
Restricted Affectivity	0.21	1.24	0.86	1.79	.255
Withdrawal	0.39	1.47	1.01	2.15	.044
Gender ^a^	−0.30	0.74	0.44	1.24	.259

*n* = 428

*CI* Confidence interval, *LL* Lower limit, *UL* Upper limit, *OR* Odds ratio, *Sure choice amount* amount of money offered as sure choice in the faith game

^a^ This variable is dummy-coded (0 for female, 1 for male)

## Discussion

The present study assessed whether the maladaptive personality trait antagonism and the maladaptive trait facet suspiciousness, as conceptualized in the AMPD [1] and measured by the PID-5 [25], are predictive of interpersonal dysfunction in the presence of specific situational affordances. Specifically, we assessed whether antagonism is associated with decreased prosocial behavior in a situation that affords the possibility for exploitation (the dictator game) and whether suspiciousness is associated with decreased trusting behavior in a situation comprising dependence under uncertainty (the faith game).

Supporting hypothesis 1, the level of antagonism was positively associated with the amount of money participants extracted in the dictator game [10, 11]. Thus, higher levels of antagonism were associated with lower levels of prosocial behavior or, framed differently, greater exploitation of interaction partners. A one unit increase in antagonism was accompanied by an increase of approximately 2€ of money extracted in the dictator game. In an additional exploratory analysis, we tested the incremental effect of antagonism beyond all other PID-5 traits and were able to replicate this result. Only antagonism showed a significant positive association with dictator game extractions, whereas none of the other PID-5 traits (detachment, negative affectivity, psychoticism, disinhibition) did. Importantly, the effect observed in this model was comparable in size to the antagonism effect we observed in the confirmatory analysis. In sum, these findings underline the specific and selective importance of the antagonism trait for predicting prosocial behavior in situations that afford the possibility for exploitation. The finding replicates and extends previous findings on increased dictator game extractions in categorical PDs characterized by heightened antagonism, such as antisocial PD (e.g., [8, 9]).

In sum, the present findings provide reason to assume that individuals with high levels of antagonism may be prone to take advantage of situations in which they can exploit others, which may also occur in the therapeutic context. For instance, in group settings or inpatient care, where patients regularly have to take on responsibilities for and cooperate with the group, those high in antagonism may exploit others to evade unwanted activities or duties. This can have substantial negative effects for the group dynamic, the course of the patient’s own treatment, and that of others. Patients high in antagonism are likely to show this exploiting behavior with little insight into the potential consequences of it and may repeatedly experience rejection by others who feel exploited by them. This could lead to them being isolated from the rest of the group and disrupt the therapeutic progress. Naturally, the same behavior can also occur in interaction with the therapist and likely occurs regularly in their daily lives. As such, it may be a central factor underlying the disruption or rupture of interpersonal relationships. Clinicians should thus be mindful of the increased likelihood of individuals high on antagonism showing exploiting tendencies (without assuming that everyone who scores high on this trait *will* show this behavior) and address them once they occur. Specifically, therapists could aim to help patients understand how others react to them when they behave in an exploiting way, in order to decrease interpersonal problems.

In addition to antagonism, the present study examined the association between the detachment facet suspiciousness and trusting behavior shown in the faith game [23], an economic game that models the situational affordance of dependence under uncertainty. Contrary to our hypotheses, we did not observe a positive association between suspiciousness and the likelihood of selecting the sure choice in the faith game. In exploratory analyses, we tested whether any of the other facets of detachment (beyond suspiciousness) predicted faith game performance, and observed no significant effects after correcting for multiple testing. Likewise, we tested whether any of the PID-5 traits predicted faith game outcomes. Here, too, no association reached statistical significance. This led us to further probe whether the experimental manipulation we implemented in the faith game was successful, as an unsuccessful manipulation could explain the lack of *any* associations with the maladaptive traits that we observed.

First, we inspected the main effect of the sure choice amount and found that it was not related to the likelihood of selecting the sure choice, whereas we intended the sure choice to become more appealing, the more money we offered as the sure choice. At first glance, this raises the question whether the experimental manipulation was successful and whether participants understood the game as intended. We would argue that the manipulation was still successful as, descriptively (see Table 3), the proportion of participants who selected the sure choice increased with the sure choice amount, even though this effect was not significant in the linear model. It is possible that the range of the amount offered as the sure choice was too small and that the lack of a significant main effect of the sure choice amount is ultimately a power issue. We chose to implement a moderate range of pay-offs, expecting that extreme sure choice amounts (e.g., 0.50€ or 5€) would result in almost everyone/ no one in that group selecting the sure choice. At the same time, this artificially restricted the range of the predictor variable, which may have resulted in the non-significant effect in the linear model. Future studies should consider sampling a larger range of sure choice payoffs, including the extreme ends of this distribution. Additionally, and again considering the descriptive results, varying the amount in 50 Cent steps may have resulted in units too small to be meaningful for participants. Participants in the 2.50€, 3€, and 3.50€ sure choice conditions showed fairly similar patterns in terms of the percentage of choices. Thus, the monetary difference between these levels may simply not have been meaningful to participants in a real-life context. Consequently, a manipulation of the sure choice in 1€ steps would likely be preferable in future studies. Moreover, it is possible that the payoffs that were offered were generally too low to be relevant to participants and that higher stakes are necessary. We implemented smaller payoffs to be able to draw a higher number of winners, but (due to limited funding) participants still only had a ten-percent chance of actually winning the money. Future studies with fully incentivized games and higher stakes would be ideal.

In an exploratory analysis, we further probed participants’ decisions in the faith game, depending on the amount of money they expected to constitute the faith choice (i.e. participants’ guesses about the amount the other player left for them). We observed that participants were more likely to select the faith choice, the higher the amount they expected to receive. This underlines the validity of the faith game. Additionally, we observed a significant interaction between the amount of money offered as the sure choice and participants’ faith choice estimates. The interaction indicated that a higher sure choice amount decreased the strength of the association between participants’ guesses about the faith choice amount and their likelihood of choosing it. This suggests that, when the amount of the sure choice is small, the faith choice is more attractive and the guess about the amount of the faith choice becomes more relevant. However, when the safe choice envelope contains larger amounts, the expected amount of the faith choice becomes less important.

In addition to the possibility that the monetary stakes were too small and restricted in range, it is possible that restricted variance in suspiciousness scores contributed to the absence of the expected effects. However, the distribution of suspiciousness was only slightly right-skewed, and several participants showed the highest possible mean score on this facet, thus we do not deem this explanation very likely to hold. Instead, the anonymity afforded in the faith game may have rendered the situational context less relevant for the expression of suspiciousness. As described above, individuals high in suspiciousness assume that others want to specifically harm or exploit *them*. They assume that there is some quality within themselves that invokes this negative behavior from others, rather than a general assumption that others necessarily treat everyone negatively. However, in our version of the faith game, Player A does not know Player B so they cannot specifically decide to exploit them because of any specific qualities of Player B. It is possible that, in order to use suspiciousness to predict the behavior of individuals in the faith game, the game would have to involve real interactions (such that individuals interact directly with each other). Future studies could consider implementing such a version of the faith game.

Additionally, future studies with more diverse samples are needed. We acknowledge that our sample diversity was restricted with regard to nationality, migration background, education level, and gender, which limits generalizability of the results largely to women without a migration background and with a higher education. As personality disorders affect individuals of all genders in the population [32], future samples should aim for a more diverse and equally distributed sample regarding gender. Despite our efforts to recruit in clinical and diverse contexts such as Facebook groups, Instagram channels, and online forums that address LGBTIQ+ topics, only 4.25% of participants indicated a gender identity other than cis-female or cis-male. Similarly, despite our efforts to recruit widely and through different channels, only very few participants indicated that they were non-native German speakers, had a migration background or that they identified as belonging to a minority. As stated above, this strongly limits the generalizability of our findings and replication in ethnically and racially diverse samples is needed. In contrast to these limitations to the generalizability of results, we successfully recruited participants of a wide age range, with different psychopathologies, various income levels, and occupations.

## Conclusion

In this pre-registered online study, we assessed associations between the maladaptive personality trait antagonism and prosocial behavior and between the maladaptive trait facet suspiciousness and trusting behavior in two partially incentivized economic games. Supporting our first hypothesis, higher levels of antagonism were associated with lower levels of prosocial behavior in the dictator game, which affords the situational possibility for exploitation. In exploratory analyses, this effect replicated when testing antagonism against the other four maladaptive traits, underlining the specificity of this effect to antagonism. Contrary to hypotheses, we did not observe a significant association between suspiciousness and the likelihood of selecting the sure choice in the faith game, a game that models the situational affordance of dependence under uncertainty. We discussed that this may have resulted from a low range and low absolute levels of the sure choice amounts that we implemented in the faith game and from the anonymity afforded by the game, which may have precluded the expression of suspiciousness. The results underline that maladaptive personality traits correlate with actual behavior in situations that afford their expression. Future studies are needed to further investigate the specific associations between a wider range of maladaptive traits and behavior relevant to interpersonal functioning.

## Supplementary Information

**Additional file 1: Fig. S1.** Regression Plot of the Interaction Effect of the Sure Choice Amount and the Guess About the Faith Choice in the Faith Game on the Likelihood of Choosing the Sure Choice in the Faith Game, Controlling for Gender. **Table S1.** Multiple Linear Regression Analysis for H1: Effects of the Mean Score of the PID-5-SF Dimension Antagonism on Prosocial Behavior in the Dictator Game (Amount of Money Taken Away), Controlling for Gender. **Table S2.** Logistic Regression Analysis for H2-H4: Effects of the Mean Score of the PID-5 Facet Suspiciousness and the Sure Choice Amount in the Faith Game on the Likelihood of Choosing the Sure Choice, Controlling for Gender. **Table S3.** Multiple Linear Regression Analysis: Effects of the Mean Score of the PID-5 Facet Suspiciousness on the Guess About the Faith Choice in the Faith Game, Controlling for Gender. **Table S4.** Logistic Regression Analysis: Effects of the Guess About the Faith Choice and the Sure Choice Amount in the Faith Game on the Likelihood of Choosing the Sure Choice in the Faith Game, Controlling for Gender.

## Data Availability

The datasets generated and/or analysed during the current study are available in the OSF repository: https://osf.io/2rvbg/.

## Abbreviations

AMPD: Alternative model for personality disorders
APA: American Psychiatric Association
DSM-5: Fifth edition of the diagnostic and statistical manual of mental disorders
ICD-11: Eleventh edition of the international statistical classification of diseases and related health problems
LGBTIQ+: Lesbian gay bisexual transgender intersex and queer
PD: Personality disorder
PID-5: Personality inventory for DSM-5
PID-5-SF: Personality inventory for DSM-5 (short form)
WHO: World Health Organization

## Footnotes

1 See Table 1 for detailed data on gender identities. Due to the small number of participants identifying with a gender other than cis/trans male/female, we decided not to add an additional dummy for this group. However, when including these participants and adding a second dummy variable to the model, results replicated (see supplements S1-S4).
