# Supplementary material for: Maladaptive personality traits as predictors of prosocial and trusting behavior in two economic games
Source: Borderline Personal Disord Emot Dysregul. 2022 Nov 10;9:32. doi: 10.1186/s40479-022-00201-0 (PMC9648038; doi:10.1186/s40479-022-00201-0)
Supplement: Supplementary file 1 — Additional file 1: Fig. S1. Regression Plot of the Interaction Effect of the Sure Choice Amount and the Guess About the Faith Choice in the Faith Game on the Likelihood of Choosing the Sure Choice in the Faith Game, Controlling for Gender. Table S1. Multiple Linear Regression Analysis for H1: Effects of the Mean Score of the PID-5-SF Dimension Antagonism on Prosocial Behavior in the Dictator Game (Amount of Money Taken Away), Controlling for Gender. Table S2. Logistic Regression Analysis for H2-H4: Effects of the Mean Score of the PID-5 Facet Suspiciousness and the Sure Choice Amount in the Faith Game on the Likelihood of Choosing the Sure Choice, Controlling for Gender. Table S3. Multiple Linear Regression Analysis: Effects of the Mean Score of the PID-5 Facet Suspiciousness on the Guess About the Faith Choice in the Faith Game, Controlling for Gender. Table S4. Logistic Regression Analysis: Effects of the Guess About the Faith Choice and the Sure Choice Amount in the Faith Game on the Likelihood of Choosing the Sure Choice in the Faith Game, Controlling for Gender. [file 40479_2022_201_MOESM1_ESM.docx]

## Supplement Materials

## Figure S1

*Regression Plot of the Interaction Effect of the Sure Choice Amount and the Guess About the Faith Choice in the Faith Game on the Likelihood of Choosing the Sure Choice in the Faith Game, Controlling for Gender*


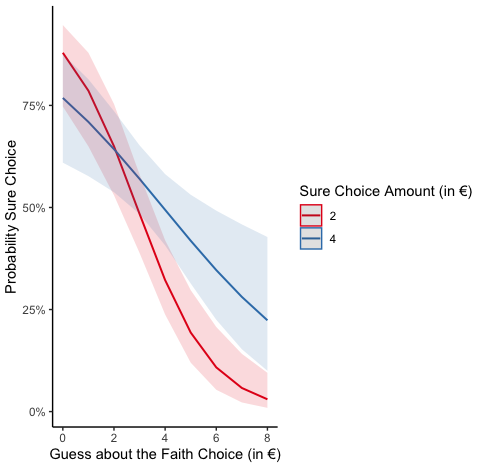


*Note.* The error bands display the 95% confidence intervals. Only minimum and maximum of the amount of money offered as sure choice are plotted here (2€ and 4€).

## Table S1

*Multiple Linear Regression Analysis for H1: Effects of the Mean Score of the PID-5-SF Dimension Antagonism on Prosocial Behavior in the Dictator Game (Amount of Money Taken Away), Controlling for Gender*

| Effect | *b* | *SE* | *t* | β | 95 % CI | | *p* |
| --- | --- | --- | --- | --- | --- | --- | --- |
|  |  |  |  |  | *LL* | *UL* |  |
| Intercept | 1.67 | 0.19 | 8.87 |  |  |  |  |
| Antagonism | 2.05 | 0.25 | 8.11 | .36 | 0.27 | 0.45 | < .001 |
| Gender_1 ^a^ | 0.36 | 0.25 | 1.46 | .07 | -0.02 | 0.15 | .144 |
| Gender_2 ^b^ | 0.57 | 0.52 | 1.10 | .05 | -0.04 | 0.14 | .274 |

*Note. n* = 445. SE = standard error; CI = confidence interval; *LL* = lower limit; *UL* = upper limit. *R^2^* = 0.14. *F*(3, 441) = 24.08, *p* < .001. ^a^ This variable is dummy-coded (0 for female and other, 1 for male). ^b^ This variable is dummy-coded (0 for female and male, 1 for other).

## Table S2

*Logistic Regression Analysis for H2-H4: Effects of the Mean Score of the PID-5 Facet Suspiciousness and the Sure Choice Amount in the Faith Game on the Likelihood of Choosing the Sure Choice, Controlling for Gender*

| Effect | Estimate | *OR* | 95 % CI | | *p* |
| --- | --- | --- | --- | --- | --- |
|  |  |  | *LL* | *UL* |  |
| Intercept | 0.03 | 1.03 | 0.83 | 1.28 | .775 |
| Suspiciousness | 0.10 | 1.10 | 0.84 | 1.45 | .471 |
| Sure choice amount | 0.10 | 1.10 | 0.85 | 1.44 | .470 |
| Suspiciousness × sure choice amount | 0.11 | 1.12 | 0.78 | 1.60 | .550 |
| Gender ^a^ | -0.25 | 0.78 | 0.49 | 1.24 | .297 |
| Gender ^b^ | -0.20 | 0.82 | 0.30 | 2.22 | .696 |

*Note. n* = 445. CI = confidence interval; *LL* = lower limit; *UL* = upper limit; *OR* = odds ratio; Sure choice amount = amount of money offered as sure choice in the faith game. ^a^ This variable is dummy-coded (0 for female and other, 1 for male). ^b^ This variable is dummy-coded (0 for female and male, 1 for other).

## Table S3

*Multiple Linear Regression Analysis: Effects of the Mean Score of the PID-5 Facet Suspiciousness on the Guess About the Faith Choice in the Faith Game, Controlling for Gender*

| Effect | *b* | *SE* | *t* | β | 95 % CI | | *p* |
| --- | --- | --- | --- | --- | --- | --- | --- |
|  |  |  |  |  | *LL* | *UL* |  |
| Intercept | 3.76 | 0.18 | 21.09 |  |  |  |  |
| Suspiciousness | -0.32 | 0.13 | -2.46 | -.12 | -0.21 | -0.02 | .014 |
| Gender ^a^ | -0.42 | 0.22 | -1.88 | -.09 | -0.18 | 0.00 | .061 |
| Gender ^b^ | -0.50 | 0.47 | -1.06 | -.05 | -0.14 | 0.04 | .290 |

*Note. n* = 445. SE = standard error; CI = confidence interval; *LL* = lower limit; *UL* = upper limit. *R^2^* = 0.02. *F*(3, 441) = 3.28, *p* = .021. ^a^ This variable is dummy-coded (0 for female and other, 1 for male). ^b^ This variable is dummy-coded (0 for female and male, 1 for other).

## Table S4

*Logistic Regression Analysis: Effects of the Guess About the Faith Choice and the Sure Choice Amount in the Faith Game on the Likelihood of Choosing the Sure Choice in the Faith Game, Controlling for Gender*

| Effect | Estimate | *OR* | 95 % CI | | *p* |
| --- | --- | --- | --- | --- | --- |
|  |  |  | *LL* | *UL* |  |
| Intercept | 3.35 | 28.48 | 3.58 | 266.00 | .002 |
| Faith choice guess | -1.11 | 0.33 | 0.32 | 0.58 | < .001 |
| Sure choice amount | -0.55 | 0.58 | 0.54 | 1.10 | .101 |
| Faith choice guess × sure choice amount | 0.21 | 1.23 | 1.02 | 1.47 | .022 |
| Gender ^a^ | -0.48 | 0.62 | 0.37 | 1.03 | .069 |
| Gender ^b^ | -0.52 | 0.59 | 0.20 | 1.72 | .335 |

*Note. n* = 445. CI = confidence interval; *LL* = lower limit; *UL* = upper limit; *OR* = odds ratio; Sure choice amount = amount of money offered as sure choice in the faith game; Faith choice guess = participants’ guess about the faith choice in the faith game. ^a^ This variable is dummy-coded (0 for female and other, 1 for male). ^b^ This variable is dummy-coded (0 for female and male, 1 for other).
